# From handbooks to platforms: Japan’s maternal and child health services in the digital era

**DOI:** 10.7189/jogh.15.03028

**Published:** 2025-06-20

**Authors:** Naruaki Imoto, Masaru Hayashi

**Affiliations:** 1 Advanced Research Institute for Health Science, Bunkyo Ward, Juntendo University, Tokyo, Japan; 2 Department of Obstetrics and Gynaecology, Tokai University School of Medicine, Isehara, Japan

## Abstract

The World Health Organization promotes universal health coverage and advocates digital initiatives. Japan has followed suit by pursuing the maternal and child health (MCH) system digitalisation. For decades, Japan has utilised a paper-based MCH handbook, credited with helping to maintain low perinatal mortality rates. However, such paper-based systems – both in Japan and abroad – often struggle with fragmented oversight, limited updates, and difficulty adapting to new technologies, thus fuelling demands for digital transformation. In this commentary, we describe Japan’s ongoing transition to digital MCH services and the complexities of modernising a long-established paper handbook, including the need to maintain user confidence, ensure data security, and reconcile diverse stakeholder interests. Japan’s experiences, reflecting both advantages and challenges, offer a cautionary yet instructive model for countries that digitise paper-based MCH frameworks.

The World Health Organisation (WHO) has identified the strengthening of maternal and child health (MCH) services as a key step toward achieving universal health coverage [1]. The Global Strategy on Digital Health (2020–25) underscores the potential of digital solutions to reinforce health care systems [2]. Against this backdrop, Japan, long dependent on a paper-based system for MCH services, now stands at a pivotal crossroads as it seeks to evolve into a digital ecosystem. Although the process in Japan has encountered multiple challenges, this experience may offer valuable lessons for countries undertaking similar reforms.

For over 75 years, Japan has relied on an MCH handbook as the basis of its MCH system, which includes records of prenatal and infant check-ups, medical records related to pregnancy and childbirth, vaccination records, and useful information for parents, with topics ranging from pregnancy to child-rearing. Local governments distribute this handbook to nearly all pregnant women [3,4], thereby ensuring regular prenatal check-ups, well-baby examinations, and ongoing parental guidance. Notably, Japan has enjoyed a very low perinatal mortality rate – an outcome driven by universal health coverage, high literacy rates, and a dense primary-care network, as well as the complementary but substantial role played by handbooks [3–6]. However, this paper-based system poses administrative, logistical, and technological challenges. Data about maternal and child health information remains fragmented across local authorities because over 1700 municipalities in Japan independently decide the layout, content, and printing contracts without using a centralised quality-control or version-tracking system, and continuous updates or integration with newer technologies are difficult to achieve. Moreover, only 789 municipalities (45.3%) use ‘My Number’ to share information about infant health check-ups and other services. In these municipalities, maternal and child health information is manually entered from paper-based records and stored in electronic format in the municipality’s system for use only within the municipality, resulting in fragmented information between municipalities [7].

While decades of refinement have made the handbook highly effective on paper, this very sophistication complicates large-scale digitisation. Currently, more than 1700 local governments have customised the handbooks to some extent, resulting in detailed entries tailored to the specific needs of each locality. Although this provision has facilitated the dissemination of necessary administrative and medical services for maintaining the health of pregnant women and infants [3,6], it has also created barriers to leveraging the benefits of digitalisation at the national level, such as the swift sharing of consistent information between local governments. For example, information recorded in paper-based handbooks can only be shared when presented to the parent or guardian, who owns the information, by manually entering it at local governments’ or medical institutions’ closed databases. According to a national review meeting, it can take several months for the results of infant health checkups conducted at medical institutions to be digitised by local governments and shared with recipients, due to the need to process paper documents [8]. The drawbacks of information fragmentation and the necessity for standardising information have also been reported in a survey conducted in Vietnam [9]. Furthermore, while the national government sets minimal MCH standards, each municipality makes independent decisions on content and operations, causing variability that impedes a unified digital format. Nevertheless, digitalisation offers the advantage of enabling the quick dissemination of standardised information stored in a common format among users, local governments, and medical institutions via a common platform.

Originally pioneered in Japan, the MCH handbook has been adopted in numerous regions worldwide. Some reports suggest that it broadens access to antenatal care, enhances health literacy, and facilitates consistent follow-up, with positive results reported in countries such as Indonesia and Afghanistan [3,4,10–12]. However, its direct effect on perinatal mortality remains less pronounced, underscoring the need for standardised formats and coordinated implementation to fully realise its benefits [3,10]. Digitising the handbook may help streamline data collection and improve quality assurance, especially where paper-based methods cannot be seamlessly integrated into electronic medical records.

In response to these issues, the Japanese government began digitising MCH data in 2018. By 2020, personal health records linked to the national identification system (My Number) enabled individuals to retrieve MCH check-up information online through a government portal called ‘MynaPortal’ [13,14], thus laying the groundwork for more extensive initiatives, including the eventual integration of the MCH handbook into digital platforms. In 2022, authorities further aligned data standards and tackled quality concerns, creating conditions for improved continuity of care and more data-driven policymaking. These endeavours fall under a broader national digital transformation strategy, motivated by an ageing population and declining birth rates that threaten health care financing and social security. To implement the 2022 Basic Policy on Economic and Fiscal Management and Reform, which promotes personal health records’ integration, standardised electronic health records, and robust data-sharing [15], the government has launched measures to optimise MCH data flows and strengthen maternal and child health governance.

This approach is mainly represented by the Public Medical Hub (PMH), introduced in 2023 by Japan’s Digital Agency [16]. The PMH enables municipalities and health care providers to share MCH data seamlessly. By linking with the MynaPortal or with private-sector applications through application programming interfaces, it integrates MCH handbook functions into smartphones, allowing municipalities to recommend health check-ups to their residents and request them to enter pre-consultation information and other details (**Figure 1**). This initiative eliminates the need for paper-based exchanges and creates an ecosystem that offers convenient, efficient access to MCH information. Although nationwide rollout is envisioned after fiscal year 2026, pilot projects in several municipalities currently explore how best to coordinate MCH data using the PMH. In tandem, the government is reviewing pertinent regulations, and while incorporating the opinions of diverse stakeholders, it is formulating guidelines that outline functional requirements and standard specifications for issuing electronic MCH handbooks. Discussions are under way to improve user convenience not only by digitising the current items in the maternity health record book but also by incorporating useful features offered by private maternity health apps, such as health check reminders and functions that enable communication between users, local governments, and medical institutions [7,14,17]. While it is necessary to unify the different data standards of each municipality in order to coordinate maternal and child health data through the PMH, the possibility of losing the ability to conduct municipality-specific surveys that reflect local conditions, which have been paper-based thus far, has also been discussed [8]. To address concerns that a national schema would erase locally tailored content, the PMH architecture permits each municipality to append optional survey items or guidance pages. These municipality-specific fields are stored as a flexible ‘local extension’ table that does not interfere with the core data set used for inter-municipal exchange [16].

**Figure 1:**
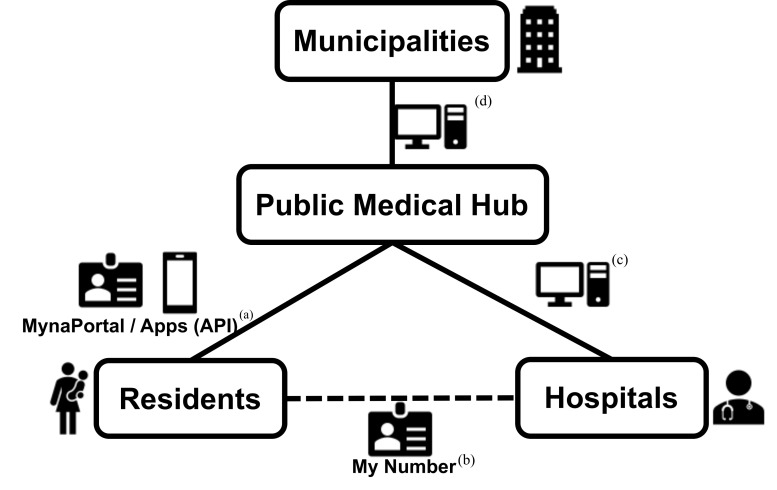
Schematic of information collaboration on maternal and child health via the Public Medical Hub (PMH) and examples of use cases. **Panel A.** Residents of the municipality use their My Number to access maternal and child health (MCH) records (eg, check-up results) online and optionally enter their medical history information before going to a hospital. API: application programming interface. **Panel B.** After providing their My Number at a hospital, the individual’s identity is verified, allowing health care providers to view existing MCH information. **Panel C.** MCH information can be reviewed, and new MCH information will be entered into the PMH by the hospital. **Panel D.** Local governments retrieve MCH data from the PMH and use it to prompt residents to undergo recommended check-ups. Maternal and child health information will be shared seamlessly when necessary, even outside the municipality of residence (for example, when shifting residences).

Despite these developments, challenges persist, and their resolution is far from guaranteed. First, the government’s efforts to accelerate the digital transformation of MCH services may have caused anxiety among people with limited internet access and those with a strong attachment to paper-based records [18]. Amid rapid digitalisation, several voices call for the continued use of paper handbooks. In a survey conducted in 2020, approximately 30% of respondents (mothers and pregnant women) said they only wanted a paper maternity health record book [19], and another study conducted in 2021 showed that >50% of the respondents preferred digital handbooks displayed on their smartphones [20]. However, according to a 2023 survey, 70% of respondents (ie, parents of infants and young children) stated that they preferred paper maternity handbooks [21], a preference that has been raised in the Japanese Diet [22]. This notable reticence is partly due to concerns about personal information leakage from My Number cards, as revealed in the first half of 2023 [23], which is discussed later in this paper. Consequently, discussions are under way regarding a gradual move away from traditional handbooks to accommodate diverse user preferences and capabilities to avoid excluding vulnerable populations [14]. Rather than suddenly switching from paper notebooks to digital ones at a certain stage, the government is considering a policy of gradually digitising paper notebooks while keeping them in use. Regular updates to paper notebooks will also be carried out under this scheme. This acknowledges the reality that digital innovation must accommodate diverse user preferences and capabilities to avoid excluding vulnerable populations.

Second, public trust in the system was eroded when multiple municipal offices mistakenly attached several individuals’ personal data to the wrong My Number identifiers in early 2023, triggering a wave of publicly reported mis-linkage incidents. In response, the Prime Minister ordered an unprecedented, cross-government review of every administrative process that relies on My Number [24]. From June to December 2023, ministries, agencies, and local governments examined 82.08 million My Number-linked records – including maternal-and-child-health files – and identified erroneous linkages in 0.01% of these records. The resulting recurrence-prevention package amends relevant legislation and issues cross-sectoral guidelines to replace the previous siloed approach and upgrades national and municipal information technology systems to enable real-time, bidirectional data verification whenever discrepancies are detected [25]. Despite these governmental measures, completely dispelling public anxiety is a challenge [26]. Because My Number underpins PMH operations, authorities must not only guarantee rigorous security protocols and multiple layers of data protection but also ensure meticulous quality control and transparent communication strategies, particularly if errors occur or personal data are compromised. Public acceptance of an identification-based digital infrastructure relies on confidence that data will remain secure and that government agencies will respond swiftly and openly to potential mishaps.

Third, effective coordination among stakeholders remains essential. The government is engaging municipal authorities, health care professionals, information technology developers, and end users in the process of digitising the maternal and child health system. However, reconciling stakeholders’ varied interests, including how to share the various human and financial costs associated with digitisation, remains a formidable challenge. Tailored dialogue, stepwise implementation, and continuous feedback loops are crucial for constructing a robust, broadly acceptable system. Without such inclusivity, even a well-designed digital tool may fail to gain traction or address local needs adequately.

If digitalisation advances successfully, the resulting system could foster more sophisticated analytics, evidence-based preventive measures, personalised interventions, and greater overall resilience in the public health sphere, thereby strengthening MCH services. However, the difficulties highlighted above – bridging the digital divide, sustaining security and trust, and harmonising varied viewpoints – must first be tackled decisively. Failing to do so risks stalling Japan’s progress in improving MCH quality and creating a truly sustainable health care framework.

Considering the abovementioned efforts in Japan and examples from other countries that use maternity health handbooks to implement maternal and child health administration, four prerequisites are always necessary for the successful digitisation of maternal and child health records. First, before procurement, the common core data set must be defined for the entire country. Adopting a single scheme will eliminate template compatibility issues between municipalities and enable population-level analysis for improving MCH. Further, the mobile front-end should be designed in collaboration with end-users, health care professionals, local governments, and vendors. Human-centred design workshops are associated with improved long-term engagement. For example, reports from Kenya showed the importance of adaptive design and implementation strategies tailored to cultural and social contexts, as well as the involvement of stakeholders, including users [27,28]. Moreover, a hybrid model must be piloted for at least one fiscal year. Maintaining paper booklets (or quick response code versions) along with the app will ensure service continuity for users who drop out of the service or use facilities with unstable communication, while also enabling data collection later. Finally, resources for cybersecurity and response measures should be secured. By allocating dedicated emergency funds and specialised staff, annual penetration testing and rapid post-incident response will be possible, as demonstrated when My Number mis-linkages were corrected in Japan.

Programmes that meet these four conditions may be better positioned to achieve long-term, nationwide scale-up outcomes that remain financially and operationally viable and to deliver equitable, high-quality MCH data across all socioeconomic strata; their true effectiveness will be assessed through Japan’s ongoing implementation. If successful, they could provide a model for other countries pursuing similar systems.

These conditions are not limited to Japan, which faces the challenges of an ageing population and low birth rates; it can also serve as a reference for developing countries that have previously utilised MCH handbooks and seek to establish MCH systems.
